# Unveiling moisture transport mechanisms in cellulosic materials: Vapor vs. bound water

**DOI:** 10.1093/pnasnexus/pgad450

**Published:** 2023-12-20

**Authors:** Yuliang Zou, Benjamin Maillet, Laurent Brochard, Philippe Coussot

**Affiliations:** Laboratoire Navier, Univ. Gustave Eiffel, ENPC, CNRS, 77420 Champs sur Marne, France; Laboratoire Navier, Univ. Gustave Eiffel, ENPC, CNRS, 77420 Champs sur Marne, France; Laboratoire Navier, Univ. Gustave Eiffel, ENPC, CNRS, 77420 Champs sur Marne, France; Laboratoire Navier, Univ. Gustave Eiffel, ENPC, CNRS, 77420 Champs sur Marne, France

**Keywords:** water transfers, cellulose-based material, magnetic resonance imaging

## Abstract

Natural textiles, hair, paper, wool, or bio-based walls possess the remarkable ability to store humidity from sweat or the environment through “bound water” absorption within nanopores, constituting up to 30% of their dry mass. The knowledge of the induced water transfers is pivotal for advancing industrial processes and sustainable practices in various fields such as wood drying, paper production and use, moisture transfers in clothes or hair, humidity regulation of bio-based construction materials, etc. However, the transport and storage mechanisms of this moisture remain poorly understood, with modeling often relying on an assumption of dominant vapor transport with an unknown diffusion coefficient. Our research addresses this knowledge gap, demonstrating the pivotal role of bound water transport within interconnected fiber networks. Notably, at low porosity, bound water diffusion dominates over vapor diffusion. By isolating diffusion processes and deriving diffusion coefficients through rigorous experimentation, we establish a comprehensive model for moisture transfer. Strikingly, our model accurately predicts the evolution of bound water’s spatial distribution for a wide range of sample porosities, as verified through magnetic resonance imaging. Showing that bound water transport can be dominant over vapor transport, this work offers a change of paradigm and unprecedented control over humidity-related processes.

**Significance Statement**

Understanding moisture transport mechanisms in cellulose-based materials is pivotal for advancing industrial processes and sustainable practices in various fields such as wood drying, paper production and use, moisture transfers in clothes or hair, humidity regulation of bio-based construction materials, etc. This study unveils the critical role of bound water transport within these fiber networks, a key factor often overlooked. By isolating diffusion processes and deriving diffusion coefficients through rigorous experimentation, we establish a comprehensive model for moisture transfer. Showing that bound water transport can be dominant over vapor transport at sufficiently low porosities, this work offers a change of paradigm and unprecedented control over humidity-related processes.

## Introduction

One of the factors considered as the most crucial in causing wear discomfort during physical activity is the presence of wetness at the skin–clothing interface (1–4). There is a similar issue with housing: the range of comfort of the user is 40–60% relative humidity (RH), which is also better for health, and an RH outside this range results in a higher demand for ventilation or heating and thus a larger energy consumption (5, 6). In this context, natural textiles (cotton, flax, hemp, jute, wool, etc.) for clothes, and bio-based construction materials, such as wood or insulation materials (fiber stacks of wheat, flax, wood, bamboo, coir, etc.), are particularly interesting as they can absorb, store, or restitute humidity, thus ensuring moisture buffering. This is mainly due to one remarkable property of these cellulose-based materials: their ability to absorb vapor thanks to hydrogen bonds, which form nanoscale water inclusions in the amorphous regions between the microfibrils of cellulosic fibers (7–10), and can represent up to 30% of the dry mass. This “bound water” is also at the origin of the swelling or shrinkage of such materials. The hair, which has a similar structure but with keratin fibrils, can absorb vapor as bound water (11, 12) to the same extent and play the same role as clothes.

Moreover, since the latent heat associated with sorption or desorption of bound water is in the same order as the huge latent heat associated with evaporation of (free) water, natural fabrics can also play a significant role in the heat loss following sweating (13), and bio-based construction materials are considered to exhibit interesting, though still not well controlled, hygrothermal properties (14, 15). Finally, bound water can offer the potential for storage and supply of large energy amounts in these materials. On another note, the extraction of bound water from such materials is an energy-intensive process. This is, for example, the case for textiles and the search for the most efficient drying technique is a critical issue (16, 17). For paper production, the extraction of the final (small) bound water fraction is the main energy consumer in the paper mill (18), with the majority of the functional properties of paper being developed during this stage (19).

However, the current description of the humidity transfers in textiles or bio-based materials relies on empirical models (17, 20–24), global laws (25–28), or statistical approaches (29), of limited applicability, for describing vapor or heat transfers through such materials. Alternatively, sophisticated models have been developed (30–38), but these models involve unverified assumptions, such as considering that water is essentially transported in the form of vapor, possibly with some exchange with bound water. In this context, the permeability to vapor transport is considered to be an intrinsic property of the material, which is estimated from basic tests of steady-state water transfers when different humidity conditions are imposed at the sample boundaries (39). Surprisingly, strongly different permeability values were found depending on the bound water content (40, 41), whereas the porous structure through which vapor diffuses does not significantly change.

It is worth noticing that, in contrast with the above approaches, for paper materials, following the pioneering and insightful work of Ramarao et al. (42–44), several works converge on the idea that the main processes of water transport are (i) vapor diffusion through the porosity (interfiber transport) and (ii) bound water diffusion inside the cellulose fiber matrix (intrafiber transport). This allowed for the development of relatively simple models (43–46). However, the diffusion coefficient values associated with each of these processes are unknown a priori, although various theoretical approaches have been proposed (45, 47). Existing (macroscopic) measurements cannot directly distinguish each transport type so that one obtains a (global) diffusivity parameter (43, 48). Its variations with the saturation (current to maximum water amount ratio) can also be interpreted to get further, but still somewhat speculative, information on the respective values of diffusion coefficients (46). Finally, a nice approach was developed to determine the specific values of each diffusivity (49) based on multiple wet-cup experiments in different RH ranges, but the form of the variation of the diffusion coefficient of bound water with the saturation had to be assumed.

Here, with a model material representative of fabric, hair, paper, or bio-based wall, we unequivocally show that bound water transport inside the network of fibers in contact plays a critical role, and even becomes dominant over vapor transport at low porosity. Then, from experiments isolating each process, we determine the diffusion coefficients associated with each transport type (vapor and bound water). This allows us to set up a general model of moisture transfer in these materials taking into account the main types of transport. Finally, we show that this model predicts very well, without any parameter fitting, the evolution of the spatial distribution of water over time as determined by magnetic resonance imaging (MRI) for the different porosities, during drying tests. This opens the way to the prediction, control, and adaptation of humidity transfer in textiles, bio-based walls, hair, or papers, allowing significant progress in advanced industrial processes, energy efficiency, and sustainable practices.

## Results

**Fig. 1.**
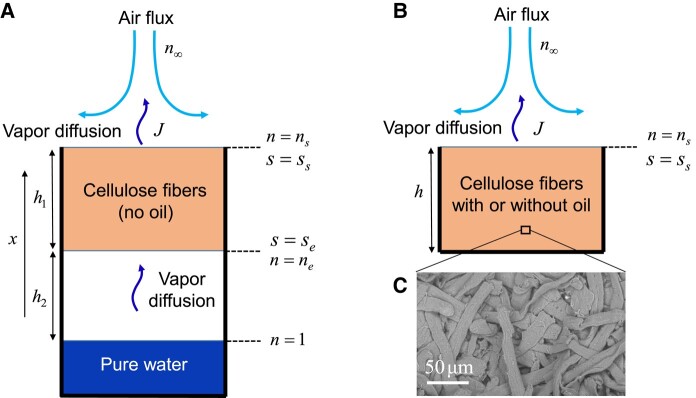
Schemes of setups for (A) steady-state diffusion through the sample and (B) for drying tests. *n* is the relative humidity in the air regions, and *s* is the moisture content, the ratio of (bound) water mass to the dry mass of the solid. (C) Scanning electron microscope image of the sample structure (porosity 0.5).

Let us consider the moisture transport through a cellulose fiber stack (see Fig. 1c). The sample is sealed over a water bath, which as a first approximation imposes an RH (noted $n_{e}$) of 1 around the sample bottom. The top surface of the sample is subjected to a constant dry air flux so that the RH ($n_{s}$) is about 0 in this region (see Fig. 1a). Due to the gradient of RH between the two regions, there is some transport of water from the bottom to the top region. We then weigh the system (water bath + sample) so as to track the water mass loss caused by evaporation from the sample and the liquid bath. Since water penetrates through the sample bottom and exits from the sample top in the form of vapor, we can describe the process with an apparent diffusion coefficient, *D*. In steady state, i.e. when the rate of mass loss is constant, there is no net mass change of the sample, and we deduce from the Fick’s law the expression for the mass flux per unit time and area through the sample:


(1) $$J=\rho_{0}D\frac{1}{h_{1}},$$

in which $h_{1}$ is the sample thickness and $\rho_{0}=0.02\text{kg}{\text{m}}^{-3}$ is the saturation vapor density at the ambient temperature $22^{\circ }\text{C}$. Note that this simple approach of boundary conditions leads to somewhat underestimate the diffusion coefficient but does not affect the following analysis about vapor transport. A more detailed analysis of the boundary conditions will be made later.

**Fig. 2.**
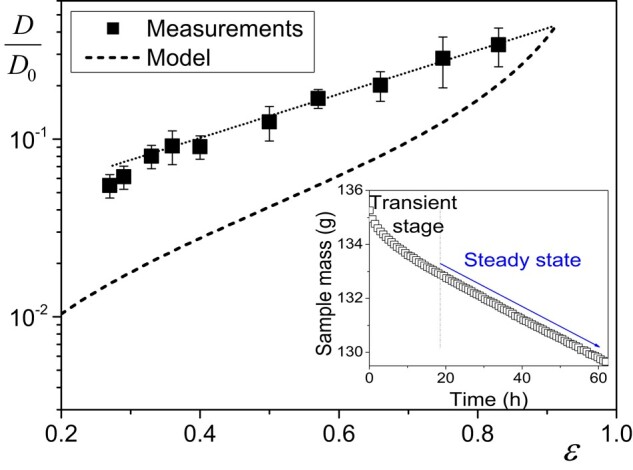
Apparent diffusion coefficient as measured from steady-state vapor flux through sample (see Fig. 1a), as a function of material porosity. The error bars were calculated from an estimation of the uncertainty on the determination of the steady-state conditions. The inset shows an example (ε=0.66) of the sample mass (including the container) variation over time with the constant rate of water extraction (steady state) reached after some time. The dotted line is a guide for the eye. The dashed line corresponds to the theoretical prediction assuming simple vapor diffusion through the porous structure (see text). Note that for this prediction, since it was not possible to make measurements in the uncompressed state, $D(\varepsilon_{0})$ was assumed to be equal to the value extrapolated from data to $\varepsilon_{0}$ (the initial porosity of the sample, i.e. before compression).

This test is reminiscent of the standard test for vapor permeability measurement (39) but here with the largest possible RH difference imposed between the two sample surfaces and the boundary condition along the sample surface controlled by applying a constant air flux (see Zou et al. (50)). The usual discussion from the standard tests concerns the impact of the RH gradient on data. Actually, we emphasize that, as a first step, we can obtain a more straightforward appreciation of the physical origin of the transport by looking at the impact of porosity (void to sample volume) on the diffusion process. Thus, we carry out the same test (with similar boundary conditions) with the same fibrous material compressed differently so as to get different porosities. The resulting values of *J* for our experiments with different sample porosities are presented in Table 1, from which one can deduce the values of *D* (see Fig. 2) through Eq. 1. *D* appears to increase by a factor of 6 when the porosity varies from 0.27 to 0.83.

**Table 1.** Characteristics of the tests with the steady-state drying setup: (from top to bottom): porosity, thickness of the air layer between the liquid bath and the sample bottom, water mass flux, and calculated RH ($n_{e}$ and $n_{s}$), respectively, along the sample bottom and top (see Fig. 1a).

ε	0.27	0.29	0.33	0.36	0.40	0.50	0.57	0.66	0.75	0.83
$h_{2}$ (cm)	2	2	2	2	2	2	1	1	1	1
*J* $\left(1{\text{0}}^{-6}\text{kg}{\text{m}}^{-2}{\text{s}}^{-1}\right)$	2.96	3.31	4.33	4.94	4.89	6.75	9.15	10.85	15.4	18.3
$n_{e}$	0.891	0.877	0.840	0.817	0.819	0.750	0.830	0.799	0.715	0.660
$n_{s}$	0.008	0.009	0.012	0.014	0.014	0.019	0.025	0.030	0.043	0.051

We can further analyze these data by comparing them with the theoretical diffusion coefficient value assuming that water is transported only in the form of vapor inside the porous medium. In that case, as long as the pore size is much larger than the mean free path of vapor molecules, the diffusion coefficient of vapor through a porous medium may be written as $\varepsilon D_{0}/\tau$ (51), in which ε is the medium porosity, *τ* is the tortuosity and $D_{0}=2.7\times 10^{-5}{\text{m}}^{2}{\text{s}}^{-1}$ is the diffusion coefficient of vapor in air. This formula precisely expresses the double impact of the presence of the solid matrix in the sample volume: the average reduction by a factor ε of the size of the paths allowed to vapor diffusion, and the average increase of the length of these paths, i.e. by a factor *τ*, for the vapor to circumvent the matrix. Thus, *τ* is the ratio of the average effective path length to the most direct path, i.e. along the sample thickness.

**Fig. 3.**
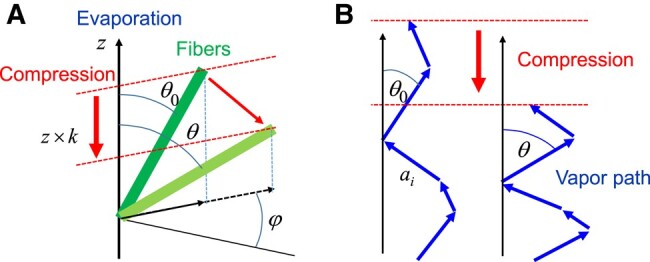
Changes of orientation of (A) a fiber from its initial to final position in a spherical coordinate frame and (B) changes of a schematic vapor path in 2D through the fiber network, resulting from a compression parallel to the evaporation axis (*z*).

It is rather difficult to predict the tortuosity of a fiber network (52), but we can circumvent this problem by focusing on the variation of *τ* with the sample porosity. More precisely, we consider a fiber stack prepared by compressing the material along one given direction by a factor *k* (final to initial thickness ratio), from some initial state of loose packing. *k* is related to the initial ($\varepsilon_{0}$) and final (ε) porosities during this compression through $k=\left(1-\varepsilon_{0}\right)/\left(1-\varepsilon \right)$. In our case, the initial state is the uncompressed state, which approximately corresponds to a porosity $\varepsilon_{0}=0.91$. The main effect under these conditions is to change the orientation of the fibers in such a way that the components of their axes along the direction of compression will be reduced by a factor *k*: $\text{cos}\theta =k\text{cos}\theta_{0}$ (see Fig. 3a). Now we define a path for vapor as some route leading from one point of the sample bottom to one point of the sample top. Because of the thermal agitation of vapor molecule, many actual paths of vapor molecules go back and forth, and we consider here only the route that provides a net progression through the sample thickness. Obviously, this route needs to circumvent the fibers, so that the distribution of paths for vapor is constrained by the presence of the solid matrix.

Let us represent such a path as a series of successive steps of distance $a_{i}$ with angle $\theta_{0,i}$ (see Fig. 3b). Under these conditions, the length of this path may be written as $L_{\mathrm{effective}}=\sum_{1<i<N_{p}}a_{i}$, while the distance covered along the main sample axis (the direction of compression and evaporation) is $L_{\mathrm{direct},0}=\sum_{1<i<N_{p}}a_{i}\text{cos}\theta_{0,i}$, which is equal to the sample thickness. The tortuosity then expresses as $\tau_{0}=L_{\mathrm{effective}}/L_{\mathrm{direct},0}$. We can consider that, essentially, we will have the same paths after compression, but now with new orientations, i.e. $\theta_{i}$, at each step, such that $\text{cos}\theta_{i}=k\text{cos}\theta_{0,i}$. This implies that the new direct length is $L_{\mathrm{direct}}=\sum_{1<i<N_{p}}a_{i}\text{cos}\theta_{i}=kL_{\mathrm{direct},0}$ and the tortuosity is now $\tau =\tau_{0}/k$.

Considering the variation of *k* with ε (see above), we finally expect the diffusion coefficient to vary as $\left[\varepsilon (1-\varepsilon_{0})/\varepsilon_{0}(1-\varepsilon )]D(\varepsilon_{0}\right)$, with $D(\varepsilon_{0})$ the diffusion coefficient for the initial porosity. This function is represented by a dashed line in Fig. 2. In fact, this is likely an overestimation since we here do not take into account the possible formation of dead-end paths during compression, implying a further increase in the length of the vapor path to circumvent these regions.

Actually, when the porosity decreases, the apparent diffusion coefficient does not decrease as expected from the simple above theory assuming only vapor transport by diffusion through the porous system: the diffusion coefficient remains significantly larger than expected, especially at low porosity (see Fig. 2). This suggests that there is another effect at the origin of water transport through the material. The only possible explanation is that the solid structure itself be able to transport water.

In fact, such an effect seems possible, since it was recently demonstrated (52) that when the fiber stack is filled with oil (leaving a thin layer free of oil along the free surface of the sample), the bound water initially contained in the fibers can be fully extracted by drying. In that case, the oil fully occupies the porosity of the sample without penetrating the fibers, so that for such oil-filled samples no vapor transport can occur, and diffusion of vapor through oil is negligible. Thus, the bound water can only be transported inside the fiber network, which means that it is not only able to diffuse along the longitudinal axis of the fiber but also to move from one fiber to another in contact with it.

**Fig. 4.**
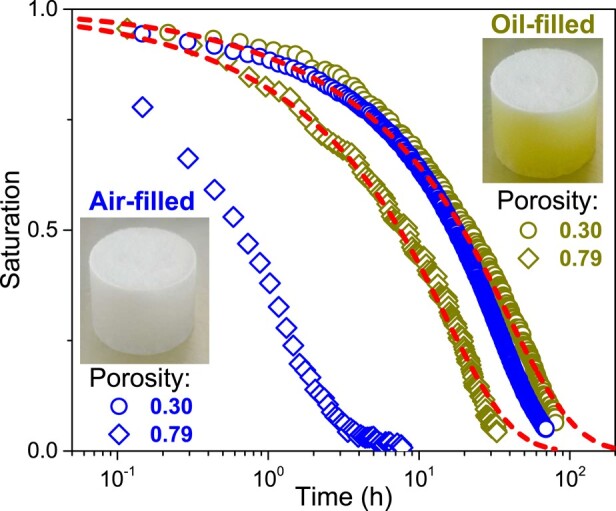
Saturation vs. time for drying tests with air-filled (blue) or oil-filled (yellow) samples at different porosities. The dotted lines correspond to a simple diffusion model with the (constant) bound water transport diffusion coefficient values shown in Fig. 6 for the corresponding porosities.

As a consequence, we have to examine again the data with standard samples in light of the possibility of water transport both by vapor diffusion through the porosity and bound water diffusion inside the fiber network. To start with, in order to appreciate qualitatively the respective role of vapor and bound water transports, we can look at the water mass evolution in the sample during transient tests. Such a situation typically occurs when one suddenly applies a dry air flux along the surface of a sample (see Fig. 1b) initially at saturation (i.e. prepared at an RH n=1). One expects the moisture to progressively leave the sample by evaporation and transport in the air flux, which ultimately leads to the full drying of the sample. Under these conditions, it is instructive to compare the results of drying tests (see Fig. 1b) carried out with the standard samples with the results obtained with samples filled with oil. In order to compare the mass evolution over times for different sample porosities, we follow the saturation, i.e. the ratio of the current to the initial water mass contained in the sample (see Fig. 4).

For a high-porosity sample (e.g. ε=0.79), the total bound water saturation for the air-filled sample decreases >10 times faster than for the oil-filled sample (see Fig. 4) suggesting that, as may be expected, vapor transport plays a major role in the air-filled sample at least over some range of saturations.

A striking result is in contrast found for a low-porosity sample (e.g. ε=0.3): the saturation vs. time curves for the oil-filled sample and the sample free of oil are very close to each other over the whole range of saturations (see Fig. 4). Firstly, this shows that, in the oil-field sample, bound water transport along the network of fibers in contact can contribute to dry the sample. Moreover, the similarity of the two drying curves for oil-filled and air-filled samples indicates that for such a low porosity, the bound water transport plays a dominant role in the water transport process for the air-filled sample, while vapor transport is almost negligible.

**Fig. 5.**
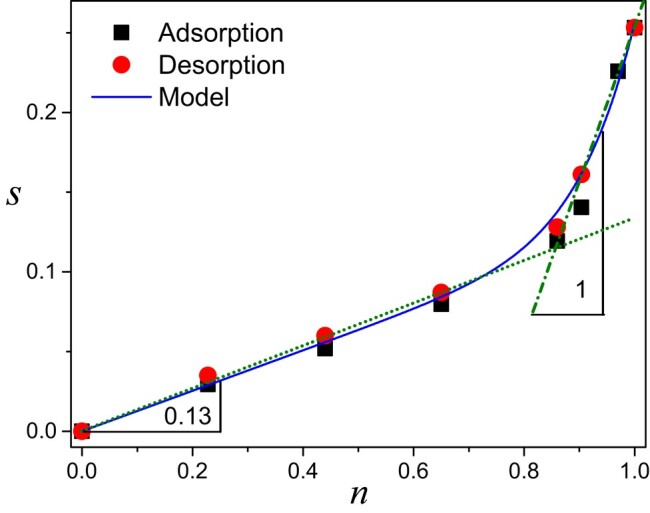
Moisture content vs. relative humidity for sorption and desorption tests. The continuous line corresponds to the equation $s=0.127\times n+{\left(n/1.227\right)}^{10}$.

Thus, we have to take into account both transport types for describing the water transport process through such hygroscopic media. As a first step, let us recall some basic concepts concerning the presence and interactions of vapor and bound water in a hygroscopic material. When a hygroscopic material such as cellulose is placed at a given relative humidity, the typical observation is, at equilibrium, a mass increase with the relative humidity, due to water absorption in the amorphous regions of fibrils (between microfibrils) (10). The moisture content (MC), i.e. the ratio of the absorbed water mass to the sample dry mass (here obtained after drying under a dry air flux), is then a function of the relative humidity at a given temperature. For our cellulose fibers, we observe (see Fig. 5) similar data for sorption, i.e. resulting from RH increase, and desorption, i.e. for RH decrease, which implies that we can describe both processes with a single sorption function s=f(n), describing the relationship between the MC value noted *s* and the RH value noted *n*, at equilibrium. This quantifies the hygroscopicity of the material. Note that these variables directly quantify the water mass, since the bound water mass per unit volume writes $\rho_{s}s$, in which $\rho_{s}=1,500\text{kg}{\text{m}}^{-3}$ is the dry cellulose density, and the water vapor mass per unit volume writes $\rho_{0}n$. Additionally, since our samples are initially saturated, we define the saturation as the ratio of *s* to its maximum possible value. At last, it is worth remarking that here *f* can be approximated within 10% by two linear functions of slope *α* equal to 0.13 up to an MC of 0.11 (i.e. n≈0.86), and equal to 1 beyond (see Fig. 5): s<0.11⇒s≈0.13n and s>0.11⇒s≈n−0.74.

For the description of the water transport through the material, the most natural way consists in assuming that there are, at each instant and in each point, diffusion of bound water due to some gradient of its concentration (i.e. the MC *s*), and diffusion of vapor due to a gradient of RH *n* (42, 44). From Fick’s law, this implies a flux of bound water $-\rho_{s}D_{b}(\partial s/\partial x)$, in which $D_{b}$ is the diffusion coefficient of bound water, and a flux of vapor $-\rho_{0}D_{v}(\partial n/\partial x)$, in which $D_{v}$ is the diffusion coefficient of vapor. In addition, as a result of the hygroscopic properties of the medium, there may be some exchange between the two phases if the local MC *s* does not exactly correspond to the equilibrium value associated with the current RH *n*. However, since the mass conservation equations for each of the water phases, i.e. vapor and bound water, contain the same term of exchange (sorption or desorption) but with an opposite sign (see Ma et al. (53)), these exchange terms do not appear in the total water mass conservation equation, which finally writes:


(2) $$\rho_{0}\varepsilon \frac{\partial n}{\partial t}+\rho_{s}(1-\varepsilon )\frac{\partial s}{\partial t}=\rho_{0}\frac{\partial }{\partial x}\left(D_{v}\frac{\partial n}{\partial x}\right)+\rho_{s}\frac{\partial }{\partial x}\left(D_{b}\frac{\partial s}{\partial x}\right).$$

This equation may be simplified by considering more precisely some physical processes. First of all, we emphasize that with such a material, under most circumstances, the MC in the fibers is very close to the equilibrium with the surrounding relative humidity, i.e. we have s≈f(n). Indeed, local equilibrium results from the water vapor molecules hitting the fiber walls and diffusing through the structure. As the rate of such collisions is very high, the limiting process is the diffusion of bound water through the thickness of the fibers. Here, the characteristic time of penetration by diffusion is $e^{2}/D_{T}$, in which *e* is the fiber thickness (say, 2μm) and $D_{T}$ is the diffusion coefficient of bound water in the direction perpendicular to the fiber surface. Assigning $D_{T}$ the order of magnitude for the diffusion coefficient along the fiber axis, i.e. $3\times 10^{-9}{\text{m}}^{\text{2}}{\text{s}}^{-1}$ (52), we find a characteristic time of diffusion in the order of $10^{-3}\text{s}$, which is obviously much shorter than the characteristic time of most processes observed in practice, and in particular of the experiments presented below. The local equilibrium, i.e. at the fiber scale, is largely reached. This finally implies that in Eq. 2 we can replace *n* by $f^{-1}(s)$. Note that this does not mean that there is a negligible exchange between the two phases.

Secondly, we remark that the first term of Eq. 2 is negligible compared to the second term of the left-hand side, since s≥0.13n (from Fig. 5) and $0.13\rho_{s}(1-\varepsilon )\gg \rho_{0}\varepsilon$. Finally, Eq. 2 rewrites


(3) $$\rho_{s}(1-\varepsilon )\frac{\partial s}{\partial t}=\rho_{0}\frac{\partial }{\partial x}\left(D_{v}\frac{\partial f^{-1}(s)}{\partial x}\right)+\rho_{s}\frac{\partial }{\partial x}\left(D_{b}\frac{\partial s}{\partial x}\right),$$

which may be expressed as a diffusion equation


(4) $$\frac{\partial s}{\partial t}=\frac{\partial }{\partial x}\left(D\frac{\partial s}{\partial x}\right),$$

with a diffusion coefficient depending on *s*: $D=\left[D_{b}(\varepsilon )+(\rho_{0}D_{v}(\varepsilon )/\rho_{s})\partial f^{-1}(s)/\partial s\right]/\left(1-\varepsilon \right)$, which may be written as


(5) $$D=\frac{1}{1-\varepsilon }\left[D_{b}(\varepsilon )+\frac{1}{\alpha }\frac{\rho_{0}D_{v}(\varepsilon )}{\rho_{s}}\right],$$

with α=∂f/∂n, the slope of the sorption curve. Note that this result is equivalent to that presented in Bandyopadhyay et al. (44) as a limiting case, but our above physical analysis shows that this should be considered as a general case.

Using the representation of the sorption curve with two straight lines (see Fig. 5) for our cellulose fibers, it follows that *α* keeps a constant value, 0.13 and 1, respectively, as long as the MC remains in a given range, i.e. either 0–0.11 or 0.11–027. Therefore, the water transport through such a hygroscopic fiber stack can be approximated as a two-step diffusion process, i.e. with two different diffusion coefficients depending on the value of the MC with respect to 0.11.

**Fig. 6.**
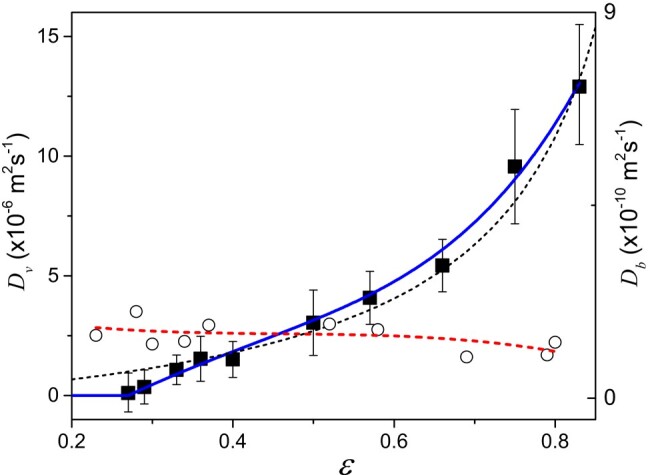
Diffusion coefficient of bound water (circles) as determined from drying tests with oil-filled samples (data of Zou et al. (52) multiplied by (1−ε)) and diffusion coefficient of vapor (squares) as deduced (see text) from steady-state transport experiments through our fiber stack samples at different porosities. The dotted line is the model ($D_{b}=(2.43-3.29\varepsilon +8.81\varepsilon^{2})(1-\varepsilon )\times 10^{-10}{\text{m}}^{2}{\text{s}}^{-1}$) fitted to bound water diffusion coefficient data and the continuous line is the model fitted to vapor diffusion coefficient data (for ε<0.27, $D_{v}=0$; for ε>0.27, $D_{v}=(15.6\times (\varepsilon -0.27)-12.9\times {\left(\varepsilon -0.27\right)}^{2}+84.9\times {\left(\varepsilon -0.27\right)}^{4})\times 10^{-6}{\text{m}}^{2}{\text{s}}^{-1}$). The error bars on the vapor diffusion coefficient data represent both the uncertainty on the wet-cup tests (see Fig. 2) and the maximum potential error associated with the discrepancy between the model (dotted line) and each data point for the bound water diffusion coefficient. The short dotted line is the model for the vapor diffusion coefficient as presented in the text.

We now have to measure the two diffusion coefficients $D_{v}$ and $D_{b}$. In a recent work (52), it was shown that the saturation vs. time data for bound water traveling inside the fiber network during the drying of an oil-filled sample may be very well fitted by a diffusion model with a single diffusion coefficient value, i.e. independent of the saturation (or equivalently, of the MC; see also Fig. 4). This coefficient must then be multiplied by (1−ε) to get the effective diffusion coefficient of bound water $D_{b}$ through our porous samples. It appears that $D_{b}$ does not vary much over the whole range of porosities, typically by a factor of 2 when the porosity varies from 0.2 to 0.8, but the data are rather scattered (see Fig. 6) due to the uncertainty of measurements. Here, in order to have a more precise estimation of this coefficient at any porosity value, we will use a model fitted to these data (see Fig. 6). This model represents the data to within 25%.

Now, we need to determine the diffusion coefficient ($D_{v}$) associated with vapor transport alone. Here, we neglect the impact, on this diffusion coefficient, of porosity variations induced by the gradient of bound water content in the fibers, as we consider this effect of second order. Note that this effect is not always negligible, and in the Materials and methods section, we further discuss how to take it into account in those cases. In this context (negligible porosity variations), considering the physical origin of $D_{v}$ as described above, $D_{v}$ is fundamentally independent of the MC, it just depends on the porosity and more generally on the geometrical structure of the porous media. In order to determine $D_{v}$, we can analyze further our initial steady-state transport experiments, now taking into account the two transport types. In addition, we will also take into account more precisely the boundary conditions.

Since for these tests the variation of the amount of water in the bath, and thus also of the level of the free surface of the bath, is negligible over the duration of the experiment, such a system imposes constant boundary conditions: n=1 along the free surface of the water bath at a constant distance from the sample bottom ($h_{2}$), and n=0 in the incoming air flux. As a consequence, after some time, a steady-state regime is reached in which the local MC s(x) and relative humidity n(x) are time-independent. Thanks to the local equilibrium, *s* is equal to the value associated to n(x) via the (equilibrium) sorption curve, represented by the equation s=f(n). In particular, along the bottom sample surface, we have $n=n_{e}$ and $s=s_{e}=f(n_{e})$, and along the top free surface of the sample, we have $n=n_{s}$ and $s=s_{s}=f(n_{s})$.

As long as *s* and *n* are in a linear portion of the sorption curve the solution of the diffusion equations for vapor and bound water transport in steady state correspond to constant concentration gradients, and there is no net transfer between the two phases since in that case n(x) and s(x) can respect the condition s=f(n). On the contrary, for *s* and *n* in the convex portion of the sorption curve a decrease of the RH *n* induces a faster reduction of the bound water diffusion, in favor of proportionally more vapor diffusion so as to keep the total diffusion flux constant. For this to happen, there is a net transfer from the bound water to the vapor (the inverse phenomenon would take place in a concave portion of the sorption curve). Note that since this transfer develops over a distance of the order of the fiber thickness it may be significant as compared to the main flux along the sample axis, even with a very small value of the difference between *s* and f(n). Thus, the sorption equilibrium assumption is still valid when one needs to estimate *s* as a function of *n* or the inverse.

In our tests, $h_{2}$ was chosen to have sufficiently low values of $n_{e}$, so that we can make the approximation of the linearity of the sorption curve over most of the sample thickness. Under these conditions, the vapor transport through the porosity of the cellulose fiber stack and the bound water transport inside the fiber network are independent. Thus, they are described by diffusion processes with diffusion coefficients respectively $D_{v}$ and $D_{b}$, and the total water flux is the sum of the two corresponding fluxes.

Finally, this provides a clear scheme of the physical processes at the different steps: vapor diffuses from the bath up to the sample bottom; at this point, a fraction of the vapor flux continues its route through the porosity up to the top surface of the sample, while the other fraction is absorbed in the cellulose fibers located along the (bottom) air–sample interface and is then transported as bound water up to the sample top via the fiber network; the vapor flux outgoing from the sample top then results from the vapor transported in the porosity and from the bound water evaporating from the fibers located along this free surface.

We deduce the expression for the total water mass flux through the sample:


(6) $$J=\rho_{0}D_{v}\frac{n_{e}-n_{s}}{h_{1}}+\rho_{s}D_{b}\frac{s_{e}-s_{s}}{h_{1}}.$$

Due to mass conservation, the simple diffusion of vapor through the homogeneous air region below the sample allows us to write another expression of this mass flux:


(7) $$\frac{\rho_{0}D_{0}}{h_{2}}\left(1-n_{e}\right)=J.$$

Finally, the mass flux can also be expressed from the diffusion process through the external air flux along the top surface of the sample. The air flux being imposed along a fixed (sample) surface (in the steady-state transport regime), the velocity can be determined from the solution of mass and momentum conservation equations. It may be shown that the local velocity is a function of the position, the details of the geometry and dimensionless numbers (54). The local vapor flux, along the sample surface, through such a velocity field, can then be expressed as $j=\rho_{0}D_{0}\left(n_{s}-n_{\infty }\right)/d$, in which $n_{\infty }$ is the relative humidity in the incoming air flux (in our case $n_{\infty }=0$) (see details in Zou et al. (50)). In this expression, the parameter *d* may be seen as an equivalent thickness of vapor diffusion; it is a function of the (dimensionless) Reynolds and Schmidt numbers (see Bergman et al. (54)), which depend only on $D_{0}$ and on a characteristic velocity of the air flux, the air density and the viscosity, and a characteristic length of the surface. For a rough interface, *d* would also depend on the roughness. The integration of this expression over the sample surface gives the total mass flux as


(8) $$J=\frac{\rho_{0}D_{0}}{\delta }n_{s},$$

in which *δ* depends on the same parameters as *d*, and thus is a constant for a given air flux.

The system of Eqs. 6–8 may be solved to find the three unknowns, $n_{s}$, $n_{e}$, and $D_{v}$. Note that *J* is determined from the sample and container mass variation in the steady-state regime (see inset of Fig. 2), and *δ* from the value of *J* in the initial stage when the sample is saturated, so that $n_{s}=1$ (see Zou et al. (50)). We carried out such experiments for samples with different porosities, and determined $D_{v}(\varepsilon )$ in the range [0.27;0.8]. The different values for *J*, $n_{e}$, and $n_{s}$ found in these tests are shown in Table 1, Materials and methods section. The resulting values for $D_{v}$ are shown in Fig. 6. Our basic theoretical prediction for $D_{v}$, i.e. $D_{v}\propto \varepsilon /1-\varepsilon$ now appears to work rather well, except at very low porosities. In order to encompass the whole range of porosities a function $D_{v}(\varepsilon )$ (see expression in Fig. 6) is fitted to these data, and will be used in the following for the theory/experiment comparison.

We can further check the validity of this model by comparing its detailed predictions in terms of spatial distribution of water inside the sample over time, for a series of drying tests (see Fig. 1b). We can quantitatively follow this process thanks to MRI, which can measure the bound water concentration along the (vertical) sample axis (see Materials and methods). Note that under almost all conditions, even if the mass transport of vapor may play a significant role, the mass of vapor is negligible compared to the bound water mass in the sample. In that case, each data point corresponds to the total (bound) water amount in a thin cross-sectional layer, and each 1D profile thus obtained describes the evolution of this value along the sample axis, at a given time after the beginning of the test. Also note that the profiles are rounded around the sample bottom, which is an artifact resulting from the constraints of MRI data treatment for such systems with a very small water content (see Materials and methods).

**Fig. 7.**
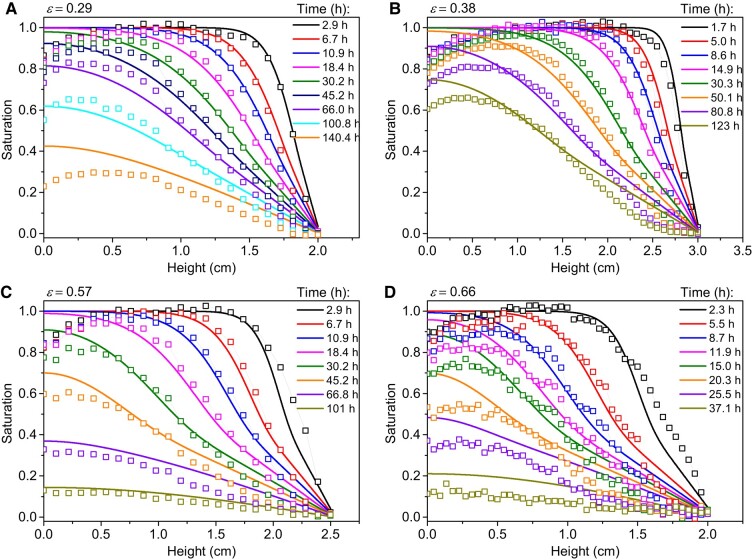
Bound water spatial distribution over time in cellulose fiber stacks of different thicknesses and porosities (see legend). The symbols correspond to experimental data obtained from MRI measurements, while the continuous lines correspond to the numerical solutions of the model (Eqs. 4 and 5) with boundary condition 8 using the parameters (diffusion coefficients) deduced from the models fitted to the data in Fig. 6.

**Fig. 8.**
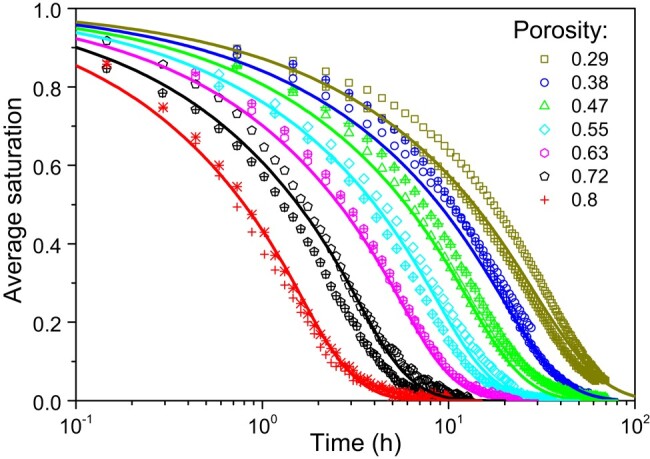
Saturation vs. time data (symbols) during drying tests of fiber stack with different porosities. The continuous lines correspond to the model (Eqs. 4–5) predictions. For each porosity value, open and crossed symbols correspond to two tests under the same conditions; the difference between the two sets of data illustrates the uncertainty on measurements.

We can then follow the draining of bound water in the sample as a function of time (see Fig. 7). The sample does not dry thanks to a dry front progressing from the free surface of the sample. Instead, the successive profiles of bound water over time are roughly similar to classical concentration distributions observed during a diffusion process (55). For example, when a saturated plane sheet is initially placed in contact with a medium which can absorb mobile species, the saturation starts to decrease abruptly around the sample top, forming a front which progresses farther into the sample and finally the level of the saturation plateau in depth starts to decrease. Moreover, the characteristic time of drying increases with the sample thickness, as evidenced by Fig. 7a and b, in which one can see that the characteristic time increases although the porosity decreases, which is due to the sample thickness increase from 2 to 3cm. At last, we can remark that, for a given sample thickness, the characteristic time of the drying test significantly increases when the sample porosity decreases (see Fig. 7a and d), i.e. from a few tens of hours for ε=0.66 to a few hundreds of hours for ε=0.29. Such trends confirm the resemblance of the phenomenon to a standard diffusion process. Finally, the dependence on porosity appears even more clearly by simply following the average saturation of the sample over time for systematic drying tests with the same thickness (0.8cm) and different porosities ranging from 0.29 to 0.8. These curves exhibit an approximately similar shape in semilogarithmic scale, shifting toward longer time as the porosity decreases (see Fig. 8). We thus deduce that the drying rate decreases significantly by a factor of approximately 20 as the porosity decreases from 0.8 to 0.29. Although other characteristics can play a significant role, the porosity may be considered as the parameter having the largest impact on the drying rate.

The models 4 and 5 with the diffusion coefficients determined above appears to predict the saturation vs. time curves very well during these drying tests over the whole range of saturations and for sample porosity ranging from 0.29 to 0.8 (see Fig. 8). We can also further check the validity of the model by looking into the details of its predictions for the spatial distribution of MC over time (see Fig. 7). It appears that these predictions are in good agreement with the data obtained from MRI measurements. Note that our model explains the particular shape of the theoretical and experimental saturation distributions in Fig. 7, which somewhat differ from the expected shape for the diffusion in a medium with homogeneous diffusion coefficient. Indeed, in contrast with the systematic concave shape of the distribution of concentration observed for simple diffusion (constant diffusion coefficient; see Crank (55)) the distributions for our cellulose systems exhibit an inflexion point situated around a saturation of 0.4. This inflexion point corresponds to the transition between the region of large diffusion coefficient ($s<0.4s_{m}\approx 0.11$, $D=\left[D_{b}+\rho_{0}D_{v}/0.13\rho_{s}\right]/\left(1-\varepsilon \right)$) and the region of low diffusion coefficient ($s>0.4s_{m}\approx 0.11$, $D=\left[D_{b}+\rho_{0}D_{v}/\rho_{s}\right]/\left(1-\varepsilon \right)$). This effect is more marked for larger porosities because in these expressions the coefficient of the second term, i.e. $D_{v}$, which is also the term varying with *s*, is larger. This implies that no general (independent of porosity) shape of the distributions can be obtained with some rescaling of the time and distance with the diffusion coefficient. Note that qualitatively similar distributions over time were recently observed by MRI in drying wood; in that case, the inflexion point separates a region containing only bound water (large diffusion coefficient) and a region containing bound and free water (low diffusion coefficient) (56).

### Dominant transport type

Finally, we can now precisely distinguish the dominant type of water transport in the hygroscopic fiber stack. Indeed, since *D* is the total diffusivity of the material for which transport is described thanks to the diffusion Eq. 4, then the total flux of mass expresses as D(∂s/∂x) which can be directly decomposed as the sum of a term $D=(D_{b}(\varepsilon )/1-\varepsilon )(\partial s/\partial x)$ corresponding to the flux of bound water, and a term $D=\frac{1}{1-\varepsilon }\frac{1}{\alpha }\frac{\rho_{0}D_{v}(\varepsilon )}{\rho_{s}}(\partial s/\partial x)$ corresponding to the flux of vapor. Both terms are proportional to (∂s/∂x). As a consequence, a direct comparison of the two terms of the diffusivity in Eq. 5 directly reflects the comparison of the different components of the mass fluxes associated with each type of process (bound water transport or vapor transport).

**Fig. 9.**
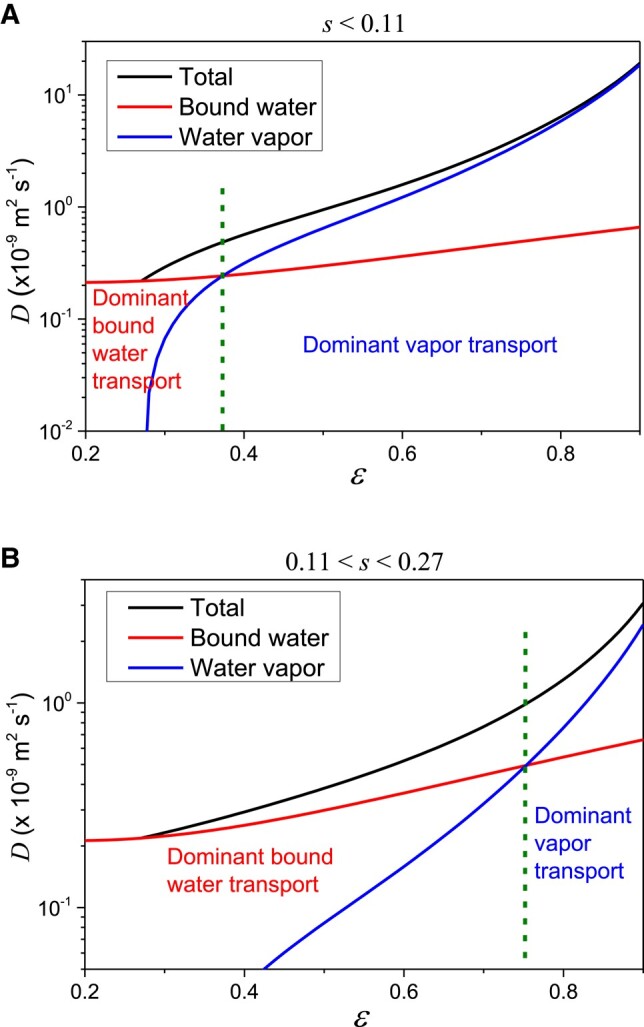
Diffusion coefficient for water transport through a hygroscopic fiber stack as a function of the porosity for two ranges of MC: (A) s<0.11 and (B) 0.11<s<0.27. The respective fractions of diffusion coefficient associated with bound water (lower curve at largest porosity) and vapor transport (intermediate curve at largest porosity) according to the two terms on the right-hand side of the expression 5 are also shown. The critical porosity for the transition between the two regimes (dominant bound water transport to dominant vapor transport) is indicated by a vertical dashed line.

Obviously, we have two different situations depending on the relative value of the MC 0.11. For s<0.11, the vapor transport is dominant over a wide range of porosities, and bound water transport becomes dominant only when the porosity reaches values below 0.37 (see Fig. 9a). On the contrary, for s>0.11, the vapor transport is dominant only for very large porosities, i.e. larger than 0.75, otherwise the bound water transport is dominant (see Fig. 9b).

**Fig. 10.**
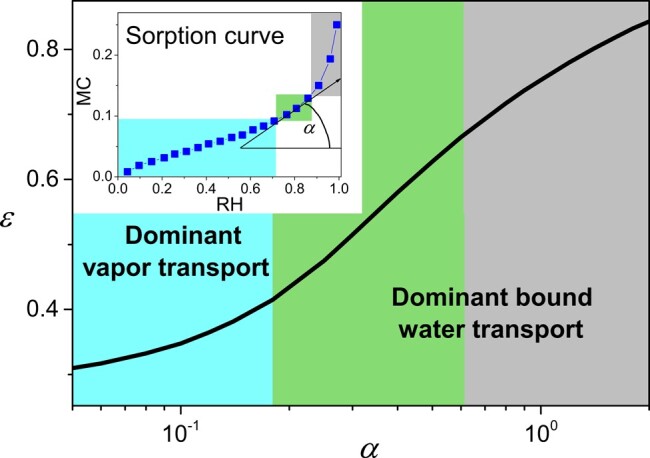
Dominant transport regime as a function of the porosity and the local slope of the sorption curve. The continuous line is the critical porosity for the transition from a bound water-dominant to a vapor-dominant transport regime as deduced from graphs analogous to those of Fig. 9 for different values of the slope of the sorption curve for the current MC value, i.e. *α* (see inset).

The above description may be generalized to other cellulose-based materials which in fact all exhibit analogous sorption curves as may for example be seen from data for a wide range of materials (57). All these curves mainly exhibit a first long part of weak slope in a wide range of *n* above 0, and a short part of steep slope at the approach of the maximum *n* (see inset of Fig. 10). Between these two regions the slope rapidly varies over a short range of *n*. Using Eq. 5 along with the expressions for the vapor and bound water diffusion coefficients determined for our cellulose stacks, we can determine, for each value of the local slope of the sorption curve (see inset of Fig. 10), the critical porosity for the transition from a bound water-dominant to a vapor-dominant regime (see Fig. 10).

We thus have a general view of the transport regimes for these hygroscopic materials (see Fig. 10). For most porosities, say above 0.4 and below 0.75 (a typical paper of $80g/m^{2}$ has a porosity of 0.47, a cotton T-shirt has a porosity about 0.65), the vapor transport is dominant when the MC is in the lower range, or more precisely, in the first part of the sorption curve, and the bound water transport is dominant when the MC is in the upper range, i.e. in the last part of the sorption curve. The short intermediate region of the sorption curve corresponds to the region of rapid variation from a vapor-dominant to bound water-dominant regime.

Thanks to this detailed knowledge of the water transport characteristics in such materials the heat transfers can be more properly described, now also taking into account the coupling between mass and heat transport due to latent heat exchange and the parameter variations with temperature. This knowledge may also be used to adjust the material properties as a function of the expected transport characteristics. In this context, it is worth reminding that for the cellulose stacks considered in this study, we described the transport properties along the direction of compression, process used for their preparation. This has a strong impact on the diffusion coefficient of bound water along the fiber network: the diffusion coefficient in the direction perpendicular to the compression direction may be up to several times larger than the diffusion in the direction parallel to the compression direction (52). In that case, we expect an extended region of dominant bound water transport toward higher porosities for low MC. Thus, it is possible to design materials with larger region of dominant bound water by adjusting the porosity and the fiber orientation. More generally, these results allow to predict exactly how the humidity transfers work through paper, textiles, fiber stacks, or hair. They for example show that even if the material porosity is very low, so that vapor transport is negligible, a relatively fast transport by bound water diffusion is in general expected when the saturation is sufficiently large. Thus, the sweat can still be rapidly evacuated through a textile saturated with bound water.

## Materials and methods

### Materials

We prepared the samples by compressing initially disordered natural cellulose fibers (Arbocel BC 200, from Kremer Pigmente) previously placed in a highly humid air (RH *n* about 97% at a temperature of $22^{\circ }\text{C}$) for several weeks. These cellulose fibers are obtained from various vegetal raw materials but it is worth noticing that our whole approach does not depend directly on the detailed origin and treatment of the fibers, but only on their measured characteristics (density, shape, sorption–desorption curve). We then investigate the transport properties along the direction of compression of the material. The cellulose fibers have a strip-like shape with an average length of 300 μm, a width of 10 μm, and a thickness about 1 μm. The dry cellulose fiber density is $\rho_{s}\approx 1,500\text{kg}{\text{m}}^{-3}$. The MC (*s*) is the bound water to dry cellulose mass ratio, and its maximum value $s_{m}$ is the MC at saturation. Here, we have $s_{m}=0.27$. We can estimate the density (*ρ*) of the cellulose fibers at an MC *s* assuming a simple mixture of dry cellulose of density $\rho_{s}$ and bound water of density close to that of bulk water, i.e. $\rho_{w}\approx 1,000\text{kg}{\text{m}}^{-3}$. We thus have $\rho =\left(1+s\right)/\left({\rho_{s}}^{-1}+s{\rho_{w}}^{-1}\right)$. The density of the saturated fibers is then $\rho_{m}=\rho (s=s_{m})$. For homogeneous cylindrical samples of cross-section area *A* and height *h*, the porosity (void to total volume ratio, noted ε) is directly deduced from ε=1−(m/ρhA), in which *m* is the sample mass. The resulting initial material porosity, i.e. with saturated fibers, of our sample ranges from 0.2 to 0.8. Note that for the estimation of the porosity, we took into account (via *h*) the slight swelling occurring when the piston is released at the end of the compression.

The sample size was 12mm diameter and 8mm thick for nuclear magnetic resonance (NMR) measurements, and 50mm diameter and between 20 and 30mm thick for MRI measurements, and 50mm diameter and 10mm thick for steady-state transport experiments.

### Drying setup

A constant dry air flux induced by a tube with relative humidity below 0.5% is imposed vertically on the sample top open surface. The dry air flux is about 2L/min for NMR measurements and 10L/min for MRI measurements with the outlet of the tube located at distances of 1cm and 3cm from the open surface, respectively. Since all other surfaces are sealed with an impermeable tape and the air flux can hardly penetrate the sample, which is a finely divided dead-end network, this setup finally essentially induces a tangential air flux along the open surface of the sample. As a consequence, the bound water can only be extracted in the form of vapor from the top open surface, which essentially leads to a 1D drying along the thickness of the sample. However, the local evaporation rate depends on the boundary layer characteristics and its vapor content which evolves along the sample surface. Thus, we expect some heterogeneity of the boundary conditions along the free surface, which might lead to some heterogeneity of sample drying in each transversal plane. Previous observations of the internal drying characteristics of clay samples or bead packings (58) indicate that such heterogeneities remain limited, probably helped by some effect of transversal equilibration of the saturation. Finally, our description of the distribution of MC along the sample axis relies on the average of MC in transversal layers, which tends to further reduce the apparent impact of such heterogeneities.

Note that the resulting drying rate, which is determined by the exact flow conditions of dry air and the shape and roughness of the sample open surface but independent of the internal material properties, can be fully characterized by a single parameter which is named the characteristic distance of vapor diffusion from the sample-open surface (see text). Here, we found, for the value of this distance δ,1mm for NMR measurements and 1.5mm for MRI measurements, as derived from the initial drying rate when the RH *n* along the sample surface is still equal to that imposed during its preparation (see Zou et al. (50)) i.e. $n_{s}=1$. The different characteristics of the tests in steady state are presented in Table 1.

### NMR and MRI measurements

The internal evolution of bound water is observed from ^1^H NMR relaxometry. The instantaneous bound water content in the cellulose sample is measured by NMR and the temporal distribution of MC along the sample during a drying test is measured by MRI. For cellulose samples 0.8−cm thick, the transverse relaxation time $T_{2}$ of bound water is observed by Bruker NMR Minispec mq20, 0.5T, 20MHz with Carr–Purcell–Meiboom–Gill sequence (59), whose main parameters are set as follows: number of scans: 256, echo time: 0.06ms, number of echos: 333, and repetition time: 2s (to get a complete relaxation of all protons). The duration of each sequence measurement is about 9min (much smaller than the characteristic drying time of our tests). A Laplace inverse transform is applied for each rephased raw signal through the Contin process (60). We follow the total bound water saturation from the integral of the signal over the whole range of $T_{2}$ distribution.

From MRI measurements, we measured the spatial distribution of the MC along the sample thickness (profile) over time. In that aim, we used a single-point imaging sequence (SPI). This sequence is dedicated to the study of materials with very short spin–spin relaxation times, generally in the range of 0.1–10 ms. Since cellulose samples contain mostly bound water whose relaxation time further decreases when the water content decreases, this sequence is well suited to follow the moisture content, even in the ultimate stages of drying. A more detailed description of the MRI sequence is provided in an earlier study (53). Here, we used a flip angle of $15^{\circ }$ with a repetition time of 200ms. The measurement time was 380 μs, of the order of the relaxation time of bound water in cellulose. The whole profiling process was repeated 256 times on purpose of phase cycling and signal accumulation, and lasted about 54min. The resolution was 0.78mm in a field of view of 5cm (corresponding to 128 slices). An inverse Fourier transform with a Gaussian filter is applied on the raw data to obtain the profiles. Note that the profiles are rounded around the sample bottom, an effect inherent to the treatment of raw MRI data relying on a Fourier Transform with the need of providing a discontinuity of signal at the sample interface. In this process, a Gaussian filter is applied to reduce the resulting noise, but this filter tends to artificially round the profiles around the interface, an effect stronger for low NMR signal, which is the case here with our samples containing only bound water. It is worth emphasizing that the procedure (in particular the filter parameters) being kept constant all along the experiment, this rounded shape remains constant (as can be seen from the profiles in Fig. 7). In order to remove this effect, a natural approach consists to rescale the profiles by the initial one; then the profiles would remain almost perfectly horizontal (around the sample bottom) for a significant duration. However, this procedure tends to introduce other sources of noise which we preferred to avoid.

### Numerical simulation details

To solve the diffusion model governing the spatiotemporal evolution of drying, an appropriate algorithm has been used. The spatial discretization is implemented by a finite element method. An implicit approximation (Backward Euler method) is adopted for the time integration in time step to guarantee the stability of the scheme. To solve the partial differential equation with high efficiency, the free time steps are taken on the basis of the convergence rate (adaptative time steps).

### Porosity variations

During our experiments, the porosity may vary along the sample axis since the fiber size (mainly its thickness) increases with its MC. Thus, we have to analyze the possible impact of this effect on our measurements and data treatments. Let us first consider the steady-state tests. Considering the slow variations of the MC in the range of RH in the sample (in these tests $n_{e}$ is smaller than, or close to 0.86, which implies that *s* was smaller than, or close to 0.11) and the limited variations of $D_{b}$ with the porosity (see Fig. 6), the maximum impact on the diffusion coefficient is 8% of variation over the sample thickness. As a consequence, the diffusion coefficient of bound water in the fiber network can be considered as approximately homogeneous, which implies that *s* indeed varies approximately linearly along the sample axis (for a steady-state diffusion process a constant diffusion coefficient implies a linear variation of the concentration, and reciprocally). It follows that, due to the sorption equilibrium and the linear approximation of the sorption curve, as long as *n* is lower than 0.86, *n* also varies linearly, and $D_{v}$ is approximately constant along *x*. Our direct observations of the apparent size of the samples during these tests confirm that it can be neglected: a shrinkage of <2% was observed.

During the drying tests, it appears that the samples nonnegligibly shrink. For the NMR tests this leads to sample thickness variations of up to 15%. As a consequence, assuming the thickness fixed to the average thickness value implies a maximum error of 8%, which will have a minor effect on the simulations (under constant porosity). Thus, in the simulations, we assumed the sample keeps a constant thickness equal to the mean thickness observed during the process. However, this shrinkage also impacts the actual porosity of the material, especially for low initial porosity, which may have a stronger impact on the transport properties. In order to take into account this effect in the simulations, we estimated the dependency of the porosity on the current local saturation and implemented this variation in the model. We assumed that the porosity variations follow a linear rule as a function of the local saturation: $\varepsilon =\varepsilon_{i}+(\varepsilon_{f}-\varepsilon_{i})\times (1-s)$, in which $\varepsilon_{i}$ and $\varepsilon_{f}$ are the (measured) porosities of the whole sample at the beginning and the end of the tests. Note that we observed a phenomenological law between the final and initial porosities, i.e. $\varepsilon_{f}=0.132+0.858\times \varepsilon_{i}$, which may be used for predictions of any case.

## Data Availability

All data are included in the manuscript and/or supporting information.
